# MicroRNAs in Pancreatic Cancer: Involvement in Carcinogenesis and Potential Use for Diagnosis and Prognosis

**DOI:** 10.1155/2015/892903

**Published:** 2015-04-19

**Authors:** Tereza Halkova, Romana Cuperkova, Marek Minarik, Lucie Benesova

**Affiliations:** ^1^Center for Applied Genomics of Solid Tumors (CEGES), Genomac Research Institute, Drnovska 1112/60, 161 00 Prague 6, Czech Republic; ^2^Internal Clinic, 1st Faculty of Medicine, Charles University and Central Military Hospital, Military Faculty Hospital, U Vojenske Nemocnice 1200, 169 02 Prague 6, Czech Republic

## Abstract

Pancreatic cancer is one of the most fatal malignancies with increasing incidence and high mortality. Possibilities for early diagnosis are limited and there is currently no efficient therapy. Molecular markers that have been introduced into diagnosis and treatment of other solid tumors remain unreciprocated in this disease. Recent discoveries have shown that certain microRNAs (miRNAs) take part in fundamental molecular processes associated with pancreatic cancer initiation and progression including cell cycle, DNA repair, apoptosis, invasivity, and metastasis. The mechanism involves both positive and negative regulation of expression of protooncogenes and tumor suppressor genes. Various miRNAs are expressed at different levels among normal pancreatic tissue, chronic pancreatitis, and pancreatic cancer and may therefore serve as a tool to differentiate chronic pancreatitis from early stages of cancer. Other miRNAs can indicate the probable course of the disease or determine the survival prognosis. In addition, there is a growing interest directed at the understanding of miRNA-induced molecular mechanisms. The possibility of intervention in the molecular mechanisms of miRNAs regulation could begin a new generation of pancreatic cancer therapies. This review summarizes the recent reports describing functions of miRNAs in cellular processes underlying pancreatic cancerogenesis and their utility in diagnosis, survival prognosis, and therapy.

## 1. Introduction

Despite the recent medical advances and new diagnostic possibilities, pancreatic cancer (PC) represents a frequent malignancy with disturbingly high mortality rates. Several histology subtypes of pancreatic tumors can be distinguished. The vast majority of them are represented by pancreatic ductal adenocarcinoma (PDAC) occurring at 96.3% of cases followed by less common cystic tumors, lymphomas, and metastases from other primary tumors [1].

The development of pancreatic cancer is associated with increasing cytological atypia forming precursor lesions, which can be divided into four stages of pancreatic intraepithelial neoplasia (PanIN I–PanIN IV) [2]. On a molecular level PanIN stages are characterized by gradual accumulation of DNA aberrations such as somatic point mutations within regulatory and coding sequences, gene amplifications, abnormal gene expressions, and allelic deletions (typically at 9p, 18q, 17p, and 6q) [3, 4]. However unlike other cancers, the detailed knowledge of molecular processes accompanying the pathogenesis of PC has not so far led to identification of a reliable biomarker for early detection of the disease. Patients occasionally exhibit elevated sedimentation of direct or indirect bilirubin, erythrocytes, and alkaline phosphatase and about a third of patients exhibit pathological glucose curve and/or anemia in case of protruding tumor [5]. Diagnostic utility of standard tumor markers is also very limited. The main markers are mucin antigens CA 19-9, CA 242, CA 50, and CA 72-4, but due to their relatively low specificity and sensitivity they are used in monitoring of disease progression rather than diagnosis [6]. Thus the standard diagnosis of PC is based on imaging techniques including an initial ultrasound followed by computed tomography (CT) or magnetic resonance imaging (MRI) and finally endoscopic ultrasound (EUS) which can definitively confirm the diagnosis, especially if the examination is supplemented by a fine needle biopsy (FNB). Unfortunately, due to the absence of specific manifestation, PC is usually diagnosed at the time of nonspecific symptoms such as fatigue, weight loss, dysorexia, abdominal pain, or jaundice (caused by compression of the duodenum) when the disease has already advanced and the prognosis of patients is very poor [5]. Only about 15% of all PC patients are diagnosed at an early stage of the disease when the tumor is operable. In these cases the tumor is surgically resected, which at present provides the only chance for cure. The chemotherapy (most often by gemcitabine) is administered after the pancreatic resection as well as in advanced inoperable stages; alternatively it is also administered in combination with radiotherapy or targeted biological therapy by erlotinib. Suppression of symptoms associated with the disease, such as biliary drainage in the duodenum or use of analgesics, is key in palliative therapy of advanced stages [7, 8]. Despite development in the management of the disease, the five-year survival is only about 5% [9].

**Figure 1:**
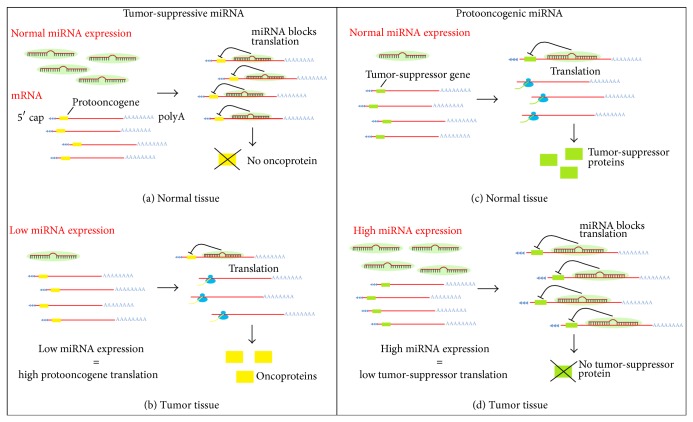
Effect of miRNAs expression on the regulation of protooncogenes and tumor-suppressor genes. Some miRNAs act as negative regulators of protooncogenes expression and therefore their role is tumor-suppression (a). In cancerous tissue, reduced levels of these tumor-suppressive miRNAs lead to increased target oncogenes promoting further tumor development (b). Other miRNAs negatively regulate expression of tumor-suppressor genes; hence, their function is (proto) oncogenic (c). In tumor tissue their increased expression results in blockage of translation of tumor-suppressors further assisting the malignant process (d).

Efforts towards finding a highly sensitive and specific tool for early diagnosis of pancreatic cancer are currently leading the clinical study of this fatal disease. The role of microRNAs in malignant transformation is gradually becoming more evident [10–12] and increased emphasis is placed on finding and testing microRNAs participating in the development of pancreatic cancer in order to improve diagnosis, assess prognosis, and design new treatment options [13, 14]. MicroRNAs (miRNAs) are endogenous noncoding short RNAs (length of 21–23 nucleotides) encoded by nuclear DNA and their main function is posttranscriptional regulation of gene expression. They bind complementarily to specific sequences of messenger RNA (mRNA), which usually leads to gene silencing via translational repression or target degradation [15, 16]. miRNAs play a crucial role in various developmental, metabolic, and cellular processes including apoptosis, cell proliferation, and differentiation. Some miRNAs regulate levels of protooncogenes or tumor-suppressor genes; therefore their expression is often altered in various tumor tissues including PC (see Figure 1). These miRNAs could serve as useful tumor biomarkers [17].

## 2. Role of miRNAs in Processes of Malignant Transformation of Pancreatic Tissue

The development of malignant transformation consists of many steps that are characterized by the disruption of various cellular processes through the damage of their control mechanisms. These are mainly faulty DNA repair system, dysfunctional cell cycle checkpoints leading to excessive cell proliferation, the failure of apoptosis, loss of contact inhibition, and cellular migration into other tissues to form distant metastases. Current reports on involvement of miRNA in pathogenesis of pancreatic cancer are mostly based on* in vitro* studies of cell lines derived from malignant cells and therefore some of the results still have to be confirmed using* in vivo* models.

### 2.1. Cell Cycle and Proliferation

Cell cycle checkpoints and kinetics are important regulators of cell proliferation. Various studies conducted in connection with PC have shown several oncogenic miRNA negatively affecting tumor-suppressor genes that act as regulators of the cell cycle progression. One of the most frequently studied miRNAs, the miR-21, affects a tumor-suppressor* PTEN* (phosphatase and tensin homologue) whose protein product prevents the proliferation of tumor cells and controls the frequency of cell division [18]. The overexpressed miR-21 attaches to the mRNA of* PTEN*, thereby reducing its tumor-suppressive function. Another potential cell cycle regulator overexpressed in PC, miR-221, affects translation of p27 (*CDKN1B* gene), a major cyclin-dependent kinase inhibitor [18]. The p27 protein binds and prevents activation of cyclin E complex with cyclin-dependent kinase 2 (CDK 2) or cyclin D complex with cyclin-dependent kinase 4 (CDK 4), therefore having a control function in progression of cell cycle in G1 phase. Also showing increased expression, miR-192 facilitates progression from G0/G1 to S phase by regulating the expression of genes involved in cell cycle control [19]. Another oncogenic miRNA, which is often overexpressed in pancreatic cancer, is miR-424-5p. It enhances the ability of cells to proliferate and migrate through downregulation of SOCS6 protein (suppressor of cytokine signaling 6) which leads to elevated ERK pathway activity [20, 21].

In an analogy to the above, in PC a lowered expression of tumor-suppressive miRNAs that regulate the efficiency of important protooncogenes can also be detected. A significant downregulation can be observed for miR-124 (miR-124-1, miR-124-2, and miR-124-3) muted as a result of promoter hypermethylation. miR-124 inhibits proliferation, invasion, and metastasis by direct interaction with the Rac1 transcript [22]. Rac1 (Ras-related C3 botulinum toxin substrate 1) is GTPase protein which is involved not only in cell cycle control, but also in cytoskeletal reorganization, activation of protein kinases, cell adhesion, epithelial differentiation, and motility [23]. Another known miRNA that contributes to tumour cell proliferation through cell cycle deregulation in PC is miR-203, whose lowered expression leads to advancement from the G1 phase [24]. Several other miRNAs, including tumor-suppressive miR-143 [25], miR-126 [26], and let-7-d [26], regulate expression of a* KRAS* oncogene, which plays a crucial role by inducing abnormal cellular proliferation through mitogen-activated protein kinase (MAPK) pathways [27].

### 2.2. DNA Repair and Apoptosis

In a normal tissue, DNA damage triggers a wide range of cellular processes resulting in either repair of the damaged sections or a programmed cell death, apoptosis. In case of abnormal function of tumor-suppressor genes or protooncogenes, however, the DNA repair pathway as well as apoptotic cascade may be completely disrupted and the cells acquire a malignant potential.

A tumor-suppressor gene* TP53* is often studied due to its major role in apoptosis and DNA repair, but it is also heavily involved in regulation of angiogenesis and cellular senescence [28]. Expression analyses have identified a number of miRNAs that contribute to* TP53* regulation. One of them, miR-34a, positively regulates apoptosis and DNA repair, while negatively altering cell cycle and angiogenesis. Levels of miR-34a are reduced to less than half or are missing completely in the tissue of pancreatic cancer compared to normal ductal epithelial tissue due to a deletion of its coding region [29–31]. It was also revealed that oncogenic miR-155 represses proapoptotic gene* TP53INP1* (tumor protein p53-inducible nuclear protein 1) which gets activated by p53. When present at high levels in PC, miR-155 increasingly prevents expression of TP53INP1, hence inhibiting apoptosis and allowing cell survival [32].

Another miRNA which negatively affects apoptosis appears to be miR-203, whose main function is inhibition of the apoptotic regulator survivin (baculoviral inhibitor of apoptosis repeat-containing 5 (BIRC5)). Downregulation of miR-203 results in increased expression of survivin, which inhibits apoptosis [24].

A recent study indicates that several miRNAs may also induce apoptosis. It can be triggered by elevated levels of miR-150^*^ and miR-630, both causing the decreased expression of transmembrane tyrosine kinase receptor IGF-1R (insulin-like growth factor 1 receptor), which has antiapoptotic properties [33].

### 2.3. Invasivity and Metastasis

The tumor cell invasivity and ability to form metastasis are an important factor that affects cancer progression. An essential step in invasivity represents the differentiation of cells through epithelial-mesenchymal transition (EMT). This means that epithelial tumor cells undergo transition to mesenchymal type, which, among others, is capable of crossing the basement membrane and entering the bloodstream. The main feature of EMT is a loss of intercellular contacts which correlates with decreased expression of the transmembrane protein E-cadherin (epithelial cadherin), an epithelial cell marker playing a key role in cell adhesion. E-cadherin (*CDH1* gene) is suppressed in several cancer types including pancreatic cancer by its repressors ZEB1 (zinc finger E-box-binding homeobox 1) and SIP1 (Smad-interacting protein 1, ZEB2, and SMADIP1) which thus act as EMT-activators [34–37]. It has been shown that members of miR-200 family (miR-200a, b, c, miR-141, and miR-429) and proteins ZEB1 and SIP1 reciprocally negatively regulate each other in a feedback loop mechanism which controls the EMT [38, 39]. Generally, the decrease in expression of miR-200 family members can trigger the epitelial to mesenchymal transition [38–43] and similar findings were published also for miR-203 [44, 45]. Most recently another miRNA, miR-208, was found to be directly involved in EMT. After overexpressing miR-208, expression of E-cadherin was decreased suggesting that miR-208 can promote the EMT [46].

Previously the involvement of miR-143 in pancreatic cancer cell invasivity was tested and its key role in regulation of Rho GTPases signaling was demonstrated [25]. Rho GTPases are G-proteins that control many processes associated with cancer metastasis formation such as cell-cell contact or cell movement. Increased activity of Rho GTPases enhances cellular invasivity and migration. miR-143 has been shown to lower Rho GTPases activity and therefore decrease levels of miR-143, which are observed frequently in pancreatic cancer cells, leading to metastatic phenotype [25].

Another miRNA involved in the formation of metastasis is miR-146a. Lower levels of mir-146a in pancreatic cancer cells were found to increase invasive behaviour [47]. Moreover it was determined that the reexpression of miR-146a can inhibit the invasivity. The molecular mechanism of this process remains unclear, but it seems to be associated with regulation of EGFR (epidermal growth factor receptor) and NF-*κ*B transcription factor signaling [47].

The opposite effect was observed for miR-10a. The expression of miR-10a promotes metastatic formation, whereas its repression leads to inhibition of invasive behavior. miR-10a supports the ability of cell to metastasize through the suppression of homeobox transcription factors HOXA1, HOXB1, and HOXB3, which, as demonstrated, may function as metastatic suppressors [48, 49].

For a summary of miRNAs involved in pancreatic carcinogenesis, which are listed in the previous text, see Table 1.

## 3. Impact of Polymorphisms and Mutations in miRNA Genes

Although changes in the coding sequences of some miRNAs such as single nucleotide polymorphisms (SNPs) and mutations may play an important role in susceptibility and development of pancreatic cancer, there are only a few studies directed at this topic. In very recent study it was shown that specific SNPs in the genes coding for precursors of miR196a2 and miR-146a may play a role in pancreatic tumorigenesis. Although significant difference between genotypes of healthy individuals and PC patients was not found, certain genetic variants of these miRNAs have been expressed more frequently in T1 and T2 stages of PC compared to T3 and T4 stages. Molecular mechanism of the different expression based on the subtle differences in sequences is currently under further study [50]. In another work a connection between mutations in miR-21 gene and pancreatic cancer was suggested [51].

## 4. miRNAs Differently Expressed in Pancreatic Tissue

### 4.1. Normal Pancreas versus Chronic Pancreatitis versus Cancer

Many research groups are engaged in analysis of aberrant expression of miRNAs in normal pancreatic tissue and pancreatic cancer. The differences are also studied and compared to data from chronic pancreatitis, which represents a major precancerous condition and an endogenous factor causing up to 16-fold increase in risk of pancreatic cancer [52]. Over the years hundreds of publications with varying levels of scientific impact have disclosed a number of miRNAs differentially expressed in all the above types of pancreatic tissues [53–75]. The most frequently reported miRNAs to be expressed at elevated levels in PDAC include miR-21 [53–57, 59, 61, 62], miR-155 [55, 58, 59, 61, 70, 72], miR-196a [58–60, 68, 72–74], miR-221 [53, 59, 75], and miR-222 [53, 59, 61, 70, 72].

### 4.2. Correlation of miRNAs Levels to PanINs

More recent papers have reported a number of miRNAs whose levels sharply change among different PanIN stages. This is typical for more advanced PanIN-2 and PanIN-3. There are currently no reports comprehensively describing specific miRNAs that correlate with each of the PanINs.

Significantly increased levels of miR-155 correlate directly with the advanced PanIN-2 and PanIN-3 [76, 77]. As mentioned earlier, the target of miR-155 is proapoptotic* TP53INP1* gene. TP53INP1 is expressed in normal as well as in the early PanIN tissues but it is lost in PanIN-3. This could be explained by the rapid growth of miR-155 in the 2nd and 3rd PanIN stages [32]. The PanIN-2 and PanIN-3 stages are supplemented by a significant increase of miR-10b [78], miR-221 [79], miR-222 [77, 79], let-7a [79], and miR-196b [77, 78] and a significant decrease of miR-217 [77, 78]. Overexpression was also observed for miR-21 [76–79] with the most notable change in PanIN-3 [76]. Also increased expression levels of miR-146a [77, 78] and miR-196a-2 [78] were found to correlate with advanced PanINs. In contrast miR-148a, whose gene is epigenetically silenced resulting in its low expression levels, correlates with early PanIN stages [80]. All miRNAs which are significantly associated with PanIN progression are listed in Table 2. In addition, a recent extensive study has revealed more than 100 miRNAs which are aberrantly expressed in different PanIN stages [77]. From them, miRNAs whose expression increases or decreases with advanced panINs are included in Table 2.

## 5. Diagnostic miRNAs

The study of miRNAs as cancer biomarkers is not restricted to tumor tissues only. More recently it was shown that almost all body fluids contain miRNA [81] as a result of either passive release from necrotic or apoptotic cells or due to an active secretion by microvesicles [82]. In comparison to mRNA, whose detection in body fluids is somewhat challenging, miRNAs are stable as they are resistant to cleavage by ribonucleases and survive extreme pH and temperature conditions [83]. With the lack of reliable approaches based on imagining techniques and/or routine tumor markers, the option of detecting miRNA in peripheral body fluids, especially blood serum, has currently a considerable potential for use in clinical practice.

Among others, miR-192 is very promising showing increased levels in serum of PDAC patients compared to healthy controls with sensitivity towards cancer at 76% and specificity at 55% [19]. Another potential biomarker is miR-18a, which occurs at high levels in tumor tissue as well as in plasma of cancer patients. In addition, miR-18a levels were found to be significantly reduced after tumor resection [84]. Other studies are focused on using combination of several circulating miRNAs to discern various stages of PC from cancer-free controls and CP [57, 85]. Recently another study introducing a promising panel of circulating miRNAs for early blood-based diagnosis of pancreatic cancer has been presented [86].

In order to increase diagnostic accuracy of early stage pancreatic cancer a combination of serum CA19-9 and quantification of miR-16 [87] or combination of CA19-9, miR-16, and miR-196a [60] may be of clinical use. The resulting sensitivity and specificity of the combined markers detected in peripheral blood for discrimination of chronic pancreatitis and pancreatic cancer are reportedly higher compared to the values for individual markers [60, 87]. Another study indicates that a combination of serum CA19-9 with the detection of the expression of miR-27a-3p from peripheral blood mononuclear cells (PBMC) can be used to diagnose pancreatic cancer with a sensitivity of 85.3% and specificity of 81.6% [69]. These tests could supposedly be applied also for screening of peripheral blood of high-risk groups. Indeed, a potential use for diagnostic purposes can be attributed to all miRNAs that are differentially expressed in PDAC compared to healthy tissue and/or chronic pancreatitis [88].

## 6. Prognostic miRNAs

Estimation of prognosis in terms of survival probability has a great significance in clinical management of pancreatic cancer. Patients often show a poor performance status and the effect of treatment is only minor. Systemic therapy or chemotherapy should therefore carefully be considered with regard to the quality of life, especially for unresectable tumors. Finding prognostic markers to assess probable course of the disease prior to treatment is therefore highly desirable. A number of literature reports are devoted to the use of miRNAs as prognostic markers. Many have demonstrated prognostic utility for miRNAs exhibiting aberrant expression in serum or in tumor tissue of PC patients. The sometimes contradicting findings are summarized in Table 3 showing the expression levels in normal tissue, in chronic pancreatitis (CP), and in cancer.

## 7. Methods for Detection of miRNA in Pancreatic Cancer

The key to a successful analysis of miRNA in pancreatic cancer is in the type, quality, and quantity of the studied sample material. As mentioned already, serum samples are best suited for clinical diagnosis and determination of prognosis. The main problem in this case, however, is in limiting amounts of miRNA as well as somewhat lower specificity. The main focus of molecular analyses, however, remains on samples acquired directly from pancreatic tissue.

Samples from surgical resections processed into frozen or paraffin sections usually provide a great amount of material for both histology as well as molecular evaluations. By the initial histology inspection presence of malignant cells is ensured prior to their subsequent molecular analysis. High content of malignant cells in the examined material is necessary for representative quantification of expression of cancer-associated genes. Many patients, however, do not undergo surgery. In such a case pancreatic tissue is only available in a form of biopsy acquired by fine needle (FNB) during an endoscopic ultrasound (EUS) examination. If the patient status permits, EUS-FNB may be performed also over the course of the disease development, to monitor the effects of therapy. The disadvantage of utilizing EUS-FNB samples for molecular examination is relatively limited amount of material, often only in a form of a cytology smear. More importantly, for EUS-FNB samples there is always an uncertainty about the actual malignant content with respect to possible contamination by blood or surrounding nonmalignant cells. Only a few of the studies directed at miRNAs rely on EUS-FNB samples [62, 67, 74, 89], while the majority use resections. An overview of the samples types used in recent literature reports on miRNAs in pancreatic cancer is summarized in Table 4.

Once the tissue sample is received, total RNA (including short RNA or miRNA) is extracted and reverse-transcribed into cDNA. Then, two alternative approaches are used (see Table 5). The first approach is based on microarray technology using hundreds to thousands of oligonucleotide hybridization probes. This approach allows screening a vast number of miRNAs in a single experiment. On the other hand miRNA array experiments require considerable amount of RNA as well as a rather sophisticated data evaluation. It is mostly used to screen for new potential miRNA biomarkers. A second approach is examination of a panel (usually up to 100) of preselected miRNAs using quantitative RT-PCR (qRT-PCR). This method is suitable, for example, to monitor aberrant levels of selected miRNAs in order to verify the context of prognosis. The qRT-PCR data are then processed by standard normalization using a set of housekeeping genes, typically including U6 [61, 62, 76, 78, 84], U44 [90], and 18S [53, 63, 70]. Normalization to other miRNAs has also been applied with miR-16 [55] and miR-54 [68].

## 8. Role of miRNA in Treatment Response and Potential for miRNA Therapy

Some of prognostic miRNAs also play a role in the efficacy of anticancer therapy and thus present themselves with new therapeutic possibilities. For example, it was found that nanomolar concentrations of antisense miR-21 and miR-221 oligonucleotides effectively inhibit their targets (oncogenic miR-21 and miR-221) and thus reduce proliferation of pancreatic cancer cell lines and, along with gemcitabine, prevent their growth [18, 91].

PDAC cells expressing elevated levels of miR-21 are chemoresistant to gemcitabine and reduce the efficiency of apoptosis induction [91, 92]. Addition of phosphoinositide 3-kinase inhibitors (PI3K inhibitors) and mTOR (mammalian target of rapamycin) serine/threonine protein kinase prevented the miR-21 (namely, the pre-miR-21) resistance, thus opening a way to gemcitabine-induced apoptosis [92]. miRNAs can be targeted, for example, by lentiviral vectors (a type of retroviruses) as recently demonstrated for miR-21, wherein PDA-derived cell lines were transduced by lentiviral vector for expression of miR-21 antagonist. Inhibition of mir-21 by its antagonist led to the cessation of tumor growth and the induction of apoptosis* in vitro* and* in vivo* (animal model) [93].

Potential drug triptolide acts on pancreatic tumor tissue as an inhibitor of cell proliferation and reduces the levels of the molecular chaperone HSP70. Rather than directly affecting HSP70 it causes increase of the levels of miR-142-3p. Ectopic expression of miR-142-3p in pancreatic tumors caused by the effect of water-soluble precursor triptolide (minnelide)* in vivo* reduces the expression of HSP70 by direct binding to the 3′UTR region of its transcript. Therefore the miR-142-3p reduces proliferation, induces cell death, and is useable as a proper target for pancreatic cancer therapies [94]. Another study has revealed a relation of miR-142-5p to the therapeutic response to gemcitabine and further states that this miRNA is an important predictive marker in patients treated with gemcitabine after tumor resection, when its higher levels indicate a longer survival [90]. Yet another therapy option comes from a possibility of recovery of function of miR-34a, a potent pro-apoptotic component involved in p53 mediated apoptosis, whose expression is reduced or lost in PDAC cells [29–31]. As shown by Ji et al. [95], restoration of miR-34a may substitute function of inactivated* TP53* gene.

miR-10a and miR-146a play important roles in pancreatic cancer invasivity and metastasis and represent potential targets for antimetastatic therapies [47, 48]. It has been shown that miR-10a promotes the metastatic behavior of PC and that its expression is regulated by retinoids [48]. The use of retinoic acid receptor antagonists inhibits miR-10a expression and stops metastasis of PDAC cells [48]. In contrast, miR-146a suppresses invasion of pancreatic cancer cells but its expression is lowered in PC compared with normal pancreatic tissue. Finally, use of isoflavones or DIM (3,3′-diinodolylmethane), both nontoxic natural compounds increasing the expression of miR-146a, also presents a promising approach to block the invasivity and metastases [47].

## 9. Conclusion

Small noncoding RNA (microRNA and miRNA) is a new hope for improvement of poor prognosis of pancreatic cancer patients. The broad involvement in cellular mechanisms of cell cycle regulation, proper functioning of DNA repair, and apoptotic control as well as mechanisms of invasivity and metastasis positions miRNAs as potential biomarkers for clinical management of pancreatic cancer. Many of the above described miRNAs are now being tested as diagnostic or prognostic markers for use in routine clinical practice. The new instrumental development in the genomic analysis facilitates their further discovery and validation. Continuing research and better understanding of the principles and complex mechanisms of miRNA-associated gene expression control may bring new possibilities for anticancer therapy of this fatal disease.

## Figures and Tables

**Table 1:** miRNAs involved in processes of malignant transformation of pancreatic tissue (↓ = reduced, ↑ = increased).

miRNA	Role of miRNA	Expression in PC cells	Impact of the aberrant expression on PC cells	Target gene	Respective references
miR-21	Oncogenic	↑	↑ proliferation and frequency of cell division	*PTEN *	[18]
miR-221	Oncogenic	↑	↑ cell cycle progression	*CDKN1B *	[18]
miR-192	Oncogenic	↑	↑ cell cycle progression	*SIP1 *and cell cycle regulatory genes	[19]
miR-424-5p	Oncogenic	↑	↑ cell proliferation and migration	*SOCS6 *	[20, 21]
miR-124	Tumor-suppressive	↓	↑ proliferation, invasion, and metastasis	*RAC1 *	[22]
miR-203	Tumor-suppressive	↓	↑ cell cycle progression, ↓ apoptosis	*BIRC5 *	[24]
			↑ epithelial to mesenchymal transition (EMT)	*CAV1 *	[45]
miR-143	Tumor-suppressive	↓	↑ cell proliferation, cellular invasivity, and migration	*GET1*,* GET2*, and* KRAS *	[25]
miR-126, let-7d	Tumor-suppressive	↓	↑ cell proliferation	*KRAS *	[26]
miR-34a	Tumor-suppressive	↓	↓ apoptosis and DNA repair, ↑ cell cycle progression and angiogenesis	*TP53 *	[29–31]
miR-155	Oncogenic	↑	↓ apoptosis	*TP53INP1 *	[32]
miR-200 family	Tumor-suppressive	↓	↑ EMT	*ZEB1*,* SIP1 *	[43]
miR-208	Oncogenic	↑	↑ EMT	*CDH1 *	[46]
miR-146a	Tumor-suppressive	↓	↑ invasivity	*IRAK-1*, *EGFR *	[47]
miR-10a	Oncogenic	↑	↑ invasivity and metastatic behavior	*HOXA1*,* HOXB1*, and* HOXB3 *	[47]

**Table 2:** miRNAs whose expression increase (↑) or decrease (↓) with increasing PanIN stages.

let-7a [79]	↑	miR-155 [32, 76, 77]	↑	miR-200a/b/c [77]	↑
miR-10b [78]	↑	miR-182 [77]	↑	miR-217 [78, 79]	↓
miR-21 [76–79]	↑	miR-183^*^ [77]	↑	miR-221 [79]	↑
miR-146a [77, 78]	↑	miR-196a-2 [78]	↑	miR-222 [77, 79]	↑
miR-148a [80]	↓	miR-196b [77, 78]	↑	miR-296-5p [77]	↓

^*^Passenger strand of pre-miRNA.

**Table 3:** Prognostic miRNAs expression (↓ reduced, ↑ increased, and ↕ comparable expression).

miRNA	miRNA level correlated with poor prognosis	Tumor versus normal tissue	Tumor versus CP tissue	CP versus normal tissue
let-7g^*^	↓ [96]			
miR-7	↓ [97]			
miR-10b	↑ [62, 98]	↑ [59, 62, 98]	↑ [59]	↑ [59]
miR-21	↑ [61, 91, 92, 99]	↑ [53–56, 59–62]	↑ [59, 60]	
miR-30d	↓ [61]			
miR-31	↓ [96]			
miR-34a	↓ [61]			
miR-122	↓ [96]			
miR-124	↓ [55]			
miR-142-5p	↑ [90]			
miR-145	↓ [99]	↑ [61]		
miR-146	↓ [99] ↑ [95]	↑ [59]		
miR-148a^*^	↓ [96]			
miR-155	↑ [70, 99]	↑ [55, 58–61, 70, 72]	↑ [59, 60]	↑ [61]
miR-187	↓ [96]			
miR-196a	↑ [58, 59]	↑ [55, 58–60, 72–74]	↕ [59] ↑ [58, 60]	↑ [59]
miR-200c	↓ [100]			
miR-203	↑ [70, 71]	↑ [70, 71]	↕ [59, 71]	↕ [71]
miR-205	↓ [97]	↑ [59]		
miR-210	↑ [70, 99]	↑ [59, 60, 70]	↑ [60]	
miR-212	↑ [96]			
miR-218	↓ [101]	↓ [101]		
miR-219	↑ [59]			
miR-221	↑ [99]	↑ [59]	↑ [59]	
miR-222	**↑** [70, 99]	**↑** [53, 59, 61, 70, 72]		
miR-675	**↑** [96]			

^*^Passenger strand of pre-miRNA.

**Table 4:** Types of samples used for miRNA analysis in pancreatic cancer.

	Serum	Fine needle biopsy	Resected tissue
Invasiveness	Low	Medium	High
Possible sampling frequency	Days	Days-weeks	Mostly no or only one sampling
The amount of sample/target miRNA	Sufficient/very small	Small/small	Sufficient/sufficient
The possibility of obtaining pure tumor tissue (cells)	No	Yes (microdissection)	Yes (macrodissection, microdissection)
Respective references	[54, 58, 68, 84–86]	Fixation [62, 67, 89] RNA stabilization solution [67, 74]	Frozen tissue [53, 56, 61, 63, 70, 72, 76, 97] Fixation [59, 67, 78, 84, 90, 96]

**Table 5:** Comparison of principal approaches for study of miRNA [102].

	qRT-PCR	miRNA microarray
Principle	PCR amplification	Hybridization
The recommended amount of RNA	10–700 ng	100–10 000 ng
Limit of detection	10–22 mol	10–18 mol
Data processing	1 day	More than 2 days
